# Trial of infographics in Northern Ireland (TINI): Preliminary evaluation and results of a randomized controlled trial comparing infographics with text

**DOI:** 10.1080/2331205X.2018.1483591

**Published:** 2018-06-11

**Authors:** Alan David McCrorie, Jingwen Jessica Chen, Ross Weller, Kieran John McGlade, Conan Donnelly

**Affiliations:** 1 School of Medicine, Dentistry, and Biomedical Sciences, Queen’s University Belfast, Belfast, United Kingdom; 2 Department of General Practice and Primary Care, Queen’s University Belfast, Belfast, United Kingdom; 3 N. Ireland Cancer Registry, Centre for Public Health, Queen’s University Belfast, Belfast, United Kingdom; 4 Finnish Institute of Occupational Health, Finland

**Keywords:** infographic, trial, evidence, communication, statistics

## Abstract

Infographics represent a potential means of improving public knowledge about cancer. However, there is little experimental evidence of their efficacy. This preliminary study investigates whether infographics are superior to text for the communication of information about cancer risk in old age via a three armed randomized controlled trial. Trial involved allocation concealment and block randomization of 30 male participants aged over 50 to receive text information (control) or one of two infographics (interventions). Participants who viewed an infographic were more likely to know the correct association between cancer risk and old age compared with those viewing text information (risk ratio = 3.0, 95% confidence interval 0.82–10.90). Participants had limited understanding of the phrases “cancer incidence” and “cancer prevalence” but good understanding of the phrases “cancer risk factor” and “cancer stage.” Possession of good numerical skills appears to be a key determinant of ability to extract meaning from statistical information provided; regardless of format. Initial results suggest icon array infographics may be more effective communication mediums than text but further study with more participants and an updated infographic is necessary to confirm this finding. Trial registration number: ISRCTN33951209.

## PUBLIC INTEREST STATEMENT

Public awareness of the link between cancer risk and getting older is low. In the United Kingdom, less than 15% of people surveyed in 2011 knew that cancer was most common in those over the age of 70 compared to those younger. This lack of awareness may be linked to poorer cancer survival due to delay in presentation to a doctor and delay in diagnosis. This study aims to address low awareness of cancer by developing and testing picture-based information resources called “infographics” that are easier to understand by the public to help make information about cancer more accessible and allow people to make more informed decisions about cancer. In this small study of 30 Northern Irish men over the age of 50 we show that infographics may be better than text for communication of information about cancer risk but further study with more people is necessary to confirm this finding.

## 1. Introduction

Infographics are a colorful and innovative means of communicating with the general public (McCrorie, Donnelly, & McGlade, 2016). A review of the cancer registry websites within Ireland and North America revealed that despite some experimentation with infographics, the majority of cancer statistics are still published in text format (British Columbia Cancer Registry, 2017; California Cancer Registry, 2017; N. Ireland Cancer Registry, 2017; National Cancer Registry Ireland, 2017; New York Cancer Registry, 2017). This is problematic because research suggests that the public struggle with some of the words and numbers used within such publications (Lipkus, Samsa, & Rimer, 2001; Peters, Smith, Funk, & Boyages, 2016; Wallsten, Budescu, Rappaport, Zwick, & Forsyth, 1986). This likely extends to some of the more specific terms used within reporting of cancer statistics such as “incidence,” “prevalence,” “risk factor,” and “stage.”

Studies indicate that infographics help the public learn (Hill et al., 2016; Zikmund-Fisher, Fagerlin, & Ubel, 2008). However, these tended to be infographics that were used to communicate personalized information such as hospital discharge instructions or adverse drug reactions. Recent research identified a public cancer knowledge gap that we wish to exploit to test infographic efficacy. Approximately 20,000 people were asked, among other things, about their awareness of the association between cancer risk and old age (Forbes et al., 2013; Simon et al., 2012). This was found to be low, particularly in Northern Ireland, where only 10.7% of adults knew cancer was most common in the elderly. Figures from the rest of the United Kingdom (13.6%), Canada (13.3%), and Australia (15.5%) were similarly poor. Cancer knowledge among citizens of other European countries tended to be much higher with 28.7% of Norwegian adults and 37.8% of Swedish adults aware of this association. The cause of the dramatic difference in knowledge between Scandinavian nations and countries within the Anglosphere is likely multifactorial and discussion of which is beyond the scope or ambition of this study.

The objectives of this study are twofold. First, to ascertain whether an icon array infographic (either alone or in combination with text) is superior to text alone for improving awareness of the association between cancer risk and old age. Second, to assess the feasibility of quantitatively measuring efficacy of icon array infographics using randomized-controlled-trial (RCT) design with a view to making any required methodological or infographic design changes prior to a second trial phase.

## 2. Methods

### 2.1. Overview

This study employed a mixed-methods approach whereby results of the RCT were quantitatively analyzed by way of odds ratios (OR) and risk ratios (RR) reporting while quotes from participants gathered during the pre-trial questionnaire phase were qualitatively analyzed via content analysis to gain an insight into baseline knowledge of some of the words and phrases used in cancer statistics reporting.

### 2.2. Interventions

**Figure 1.**
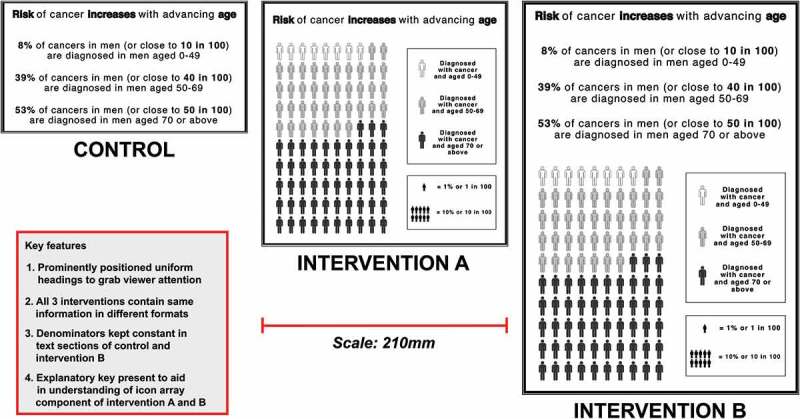
Interventions including text (control), icon array infographic (intervention A), and icon array plus text infographic (intervention B).

Data from the N. Ireland Cancer Registry. (2017)were used to produce information materials for this trial. Text information (control) was produced after a review of the content and style of statistical information available on both cancer registry websites in Ireland (N. Ireland Cancer Registry, 2017; National Cancer Registry Ireland, 2017). Choice of typeface and word count were guided by research into the area of text readability (Altpeter et al., 2015; Russell-Minda et al., 2007). Icon array was chosen as the infographic to test due to its ability to unambiguously represent part-to-whole relationships while also negating the effects of denominator neglect (Spiegelhalter, 2017; Spiegelhalter, Pearson, & Short, 2011). Two forms of infographic were produced: one which exclusively contained an icon array (intervention A) and another that contained text plus icon array (intervention B). Infographics were produced using Adobe Photoshop CC 2017 (see Figure 1). Color was omitted from infographics to mitigate the effect of color-blindness, likely present in 10% of participants (Drummond-Borg, Deeb, & Motulsky, 1989).

### 2.3. Trial methodology

This was a three-armed non-blinded RCT involving allocation concealment and computer-generated block randomization. Eligible participants (consenting males over the age of 50 who speak English) were invited to take part during separate 1-day visits to four Men Shed’s in Northern Ireland (non-profit organizations that cater for males aged 50+ by providing locations to pursue practical activities such as gardening or craftsmanship) (Milligan et al., 2015; UK Men’s Shed Association, 2017). Participants who were not eligible to participate included those under the age of 50, non-English speakers, and those who did not provide written consent.

Participants were first given a short paper-based questionnaire containing demographic questions, a 9-item statistical numeracy test previously validated in an international cross-cultural questionnaire with a Cronbach’s α (internal consistency) of 0.80, and four cancer definition questions (refer to supplementary materials) to be answered within 15 min (Galesic et al., 2010). Participants were asked to define the phrases “cancer incidence,” “cancer prevalence,” “cancer risk factor,” and “cancer stage.” Thereafter, participants were block randomized to view an A4-sized black and white image of either control, intervention A, or intervention B. Allocation was concealed by storing pre-generated assignment codes in an opaque envelope. Codes were communicated to the researcher responsible for allocating participants to their assigned intervention after questionnaire completion. Participants had a maximum of 2 min to view assigned intervention before a 30 s follow-up phase. The questionnaire had a question about the association between cancer risk and old age embedded within it. The same question was asked again during the follow-up phase.

### 2.4. Sample size

Previous research suggests that 59 participants are required for a pilot study to identify, with 95% confidence, at least one event of interest or methodological problem (Viechtbauer et al., 2015). We planned to conduct an RCT with three arms, which needed a larger sample size. Therefore, our aim was to recruit as many participants as possible to this trial. Unfortunately, due to low number of eligible volunteers willing to participate, it was not possible to obtain desired sample size.

### 2.5. Quantitative analysis

Questionnaire responses were entered into SPSS Statistics Desktop version 22.0. No participant identifiable information was collected. Descriptive summary statistics of participant demographics were expressed as mean ± standard deviation or frequency (percentage). Participant demographics were summarized and tabulated. Differences in participant demographics between RCT wings were compared using one-way analysis of variance (ANOVA) for continuous variables and chi-squared test for categorical variables. Knowledge of the association between cancer risk and old age was dichotomized (correct or incorrect) and was collected for each participant before and after viewing assigned intervention.

Unadjusted risk ratios (RR) were calculated to identify the probability of identifying the *correct* association between cancer risk and old age post-intervention. RR and 95% confidence intervals were compared between control and intervention groups. OR and 95% confidence intervals were calculated to determine the effect of numeracy score, education status, and pre-intervention cancer knowledge on post-intervention knowledge of the association between cancer risk and old age. A value of *P* < 0.05 indicates significance.

### 2.6. Qualitative analysis

Free text written responses to the four phrases participants were asked to define were analyzed using qualitative content analysis. This is a widely used method of analyzing information such as text to derive themes in psychological and sociological studies (Hsieh et al., 2005). Questionnaire responses were transcribed verbatim by two researchers working independently of each other. Both researchers cross-checked each other’s transcribed responses to clarify any misunderstandings of participant responses. Themes common to each definition were then assigned codes after data was repeatedly read in an effort to achieve data immersion. Coding was performed independently by two researchers to enhance methodological rigor. Quotes were converted into “coding units” prior to being sorted into categories and overall themes using an approach outlined in the literature (Graneheim et al., 2004). Disagreement between researchers regarding code assignment was resolved through discussion.

### 2.7. Ethics

This study was approved by the ethics committee of the School of Medicine, Dentistry, and Biomedical Sciences, Queen’s University Belfast on the 19 July 2017 (reference: 17.27v3). Trial is registered with the International Standard Randomized Control Trial Number (ISRCTN) registry (ISRCTN33951209).

## 3. Results

### 3.1. Participant demographics

**Figure 2.**
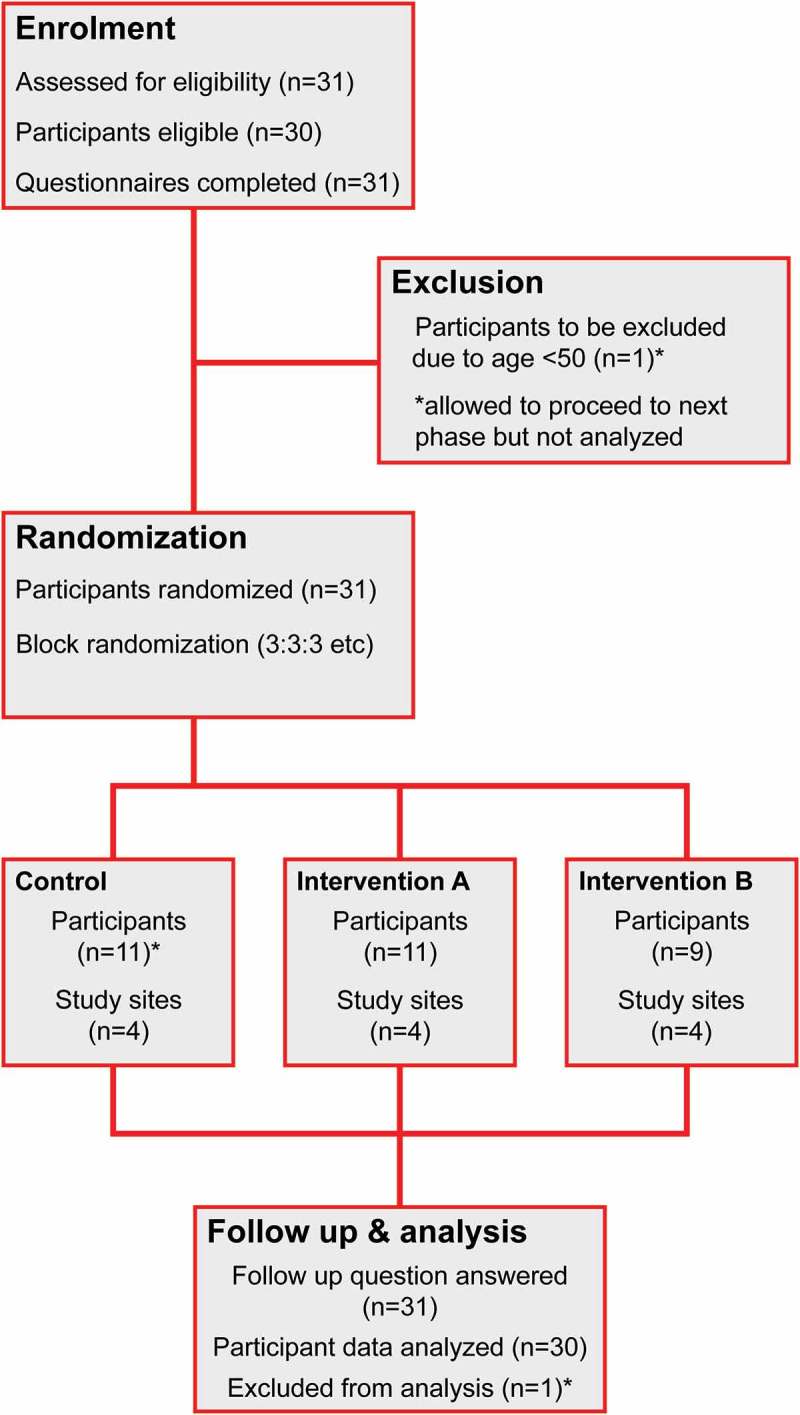
Flow diagram of participant progress through RCT where (*) indicates progress of excluded participant.

A total of 31 men agreed to participate across four study sites in Northern Ireland during July 2017. One participant was excluded from analysis for being aged less than 50 (see Figure 2). Of the remaining 30 participants, 76.7% were aged 70 or above, 36.7% had no formal educational qualifications (i.e., left school at or before age 15), 93.3% had experience of cancer, and 43.3% lived alone. With the exception of education, there were no statistically significant differences between the demographic characteristics of participants assigned to view control, intervention A, or intervention B. Participants assigned to intervention B were found to be significantly more educated than those assigned to control or intervention A (see Table 1).

**Table 1.** Participant demographics by assigned intervention

Demographic characteristics	Interventions				*P*-value
	All (*N* = 30)	Control (*N* = 10)	A (*N* = 11)	B (*N* = 9)	
**Age group (years)**					
50–69	7 (23.3)	1 (10.0)	2 (18.2)	4 (44.4)	0.224
70+	23 (76.7)	9 (90.0)	9 (81.8)	5 (55.6)	
**Education**					
No formal qualifications	11 (36.7)	6 (60.0)	5 (45.5)	0 (0.0)	0.048
At least one qualification	15 (50.0)	4 (40.0)	5 (45.5)	6 (66.7)	
Missing data	4 (13.3)	0 (0.0)	1 (9.1)	3 (33.3)	
**Cancer experience**					
Self, family, friend	28 (93.3)	10 (100.0)	9 (81.8)	9 (100.0)	1.000
None	1 (3.3)	0 (0.0)	1 (9.1)	0 (0.0)	
Missing data	1 (3.3)	0 (0.0)	1 (9.1)	0 (0.0)	
**Relationship status**					
Married or with partner	16 (53.3)	8 (80.0)	5 (45.5)	3 (33.3)	0.139
Single, divorced, widow	14 (43.3)	2 (20.0)	5 (45.5)	6 (66.7)	
Missing data	1 (3.3)	0 (0.0)	1 (9.1)	0 (0.0)	

Note: Values in parenthesis are relative frequencies in percentages based on the column total. *P*-values shown are based on Fisher’s exact test. *P* < 0.05 indicates statistical significance.

### 3.2. Effect of interventions

Baseline knowledge of the correct association between cancer risk and old age before interventions were viewed was 13.3% (*N* = 4). This increased to 46.7% post interventions (*N* = 14). Baseline knowledge among those assigned to view intervention A or B (*N* = 20) was 10% (*N* = 2). This increased to 60% post interventions (*N* = 12). When compared with text control, viewing either intervention A or B increased the probability of having knowledge of the correct association between cancer risk and old age, however this was not statistically significant (RR = 3.0 95% CI = 0.82–10.90). Viewing infographic intervention A increased probability of correctly answering the post-intervention question threefold (RR = 3.2 95% CI = 0.85–11.88) with similar effect seen in those viewing infographic B (RR = 2.8 95% CI = 0.71–10.94) (see Table 2).

**Table 2.** Effect of interventions on knowledge of association between cancer risk and old age after viewing interventions

Intervention received	Correct knowledge pre-intervention	Correct knowledge post-intervention	Risk ratio (95% confidence interval)
Control (*N* = 10)	*N* = 2	*N* = 2	1.0 (RC)
Intervention A (*N* = 11)	*N* = 2	*N* = 7	3.2 (0.85–11.88)
Intervention B (*N* = 9)	*N* = 0	*N* = 5	2.8 (0.71–10.94)
Intervention A or B (*N* = 20)	*N* = 2	*N* = 12	3.0 (0.82–10.90)

RC, reference category. Risk ratio indicates probability of having correct post-intervention knowledge.

### 3.3. Effect of other factors

The effect of numeracy, education, and baseline knowledge of cancer risk in old age on the odds of identifying the correct association between cancer risk and old age was further explored. The mean numeracy score for all participants was 4 out of 9 (median = 4, range = 1–9, IQR = 3.25). The mean score of participants who incorrectly answered the post-intervention question about cancer risk and old age was 2.94 (SD = 1.77). This compares to a mean score of 5.57 (SD = 2.10) in those who answered correctly post-intervention. The difference in scores between these two groups was highly significant (*P* < 0.001). An above average numeracy score doubled the odds of correct knowledge of cancer risk and old age post-intervention (OR = 2.1 95% CI = 1.21–3.50) (*P* = 0.008). Having at least one formal educational qualification also increased the odds of getting the post-intervention question correct (OR = 7.3 95% CI = 1.27–42.29) (*P* = 0.026) (see Table 3). The proportion of participants with at least one educational qualification who had correct knowledge of cancer risk and old age increased from 25% pre-intervention to 79% post-intervention (54% increase). This is in contrast to participants with no formal educational qualifications, of whom 36% were correct pre-intervention and 21% were correct post-intervention (15% decrease). Having correct pre-intervention knowledge of association between cancer risk and old age also increased odds of having correct post-intervention knowledge but this was not statistically significant (OR = 1.2 95% CI = 0.14–9.59) (*P* = 0.886) (see Table 3).

**Table 3.** Effect of other variables on having correct post-intervention knowledge of the association between cancer risk and old age

Variable	Odds ratio (95% confidence interval)	*P*-value
Numeracy ^a^	2.1 (1.21–3.50)	0.008
Education ^b^	7.3 (1.27–42.29)	0.026
Pre-intervention knowledge ^c^	1.2 (0.14–9.59)	0.886

a = numeracy score of >4. b = possession of ≥ 1 educational qualification. c = correct pre-intervention knowledge.

### 3.4. Cancer definition themes

Of the 30 participants who had their responses analyzed, 23 attempted to define “cancer incidence,” 19 attempted to define “cancer prevalence,” 23 attempted to define “cancer risk factor,” and 25 attempted to define “cancer stage.” Participants unaccounted for either left question space blank or did not know.

The majority of participants who attempted to define “cancer incidence” either did so using a combination of mathematical terms like “odds,” “chances,” or “numbers” or by using negative emotive phrases like “bad news,” “scare from the doctor,” or “hurt and pain.” A minority of participants (*N* = 5) seemingly confused “cancer incidence” with a diagnostic phrase such as “detection” and “diagnosis.” Similarly, the majority of participants chose to define the phrase “cancer prevalence” using either mathematical terminology or confused it with a phrase that meant increased cancer risk. While no participants gave an exact definition for either of these two phrases, those who used mathematical terms such as “percentage” appeared to have greatest insight into the meaning of these phrases.

Half of all participants (*N* = 15) chose to define “cancer risk factor” using an example of what they considered to increase the risk of cancer. These included in order of respondent frequency, “smoking,” “alcohol,” “food,” “pollution,” and “sun exposure.” A number of participants responded with more general definitions that mainly fell under the umbrella of lifestyle related risk. A small number (*N* = 3) of participants attempted to define “cancer risk factor” using probability terminology including “increased likelihood” and “increased chances.”

Three themes emerged when analyzing responses of those attempting to define “cancer stage.” The first and most numerous (*N* = 17) was the concept of growth or development as a means of defining “cancer stage.” Some participants used words and phrases associated with the concept of time such as “phase,” ‘1-2-3ʹ, and “beginning-middle-end.” Others used words or phrases describing spatial awareness such as “position,” “level,” or “localized or metastasized.” Six participants chose to further develop this theme by linking it with the concept of diagnosis or treatment by using phrases such as “early detection,” “catching cancer early,” “degree of invasion and treatability,” and “a measurement of how treatable a cancer is.” The remaining participants had little or no understanding of the term “cancer stage” and either rephrased the question or used negative emotive phrases such as “death” or “concern.”

## 4. Discussion

### 4.1. Overview

Baseline knowledge of the correct association between cancer risk and old age was comparable to that found in a recent international study (Forbes et al., 2013). A 33.4% increase (13.3% to 46.7%) in the proportion of participants aware of this association after interventions is similar to improvement achieved in other similar public health knowledge deficit interventions (Buykx et al., 2015). The increase in proportion of participants aware of the correct association is greater still when we exclusively focus on those who viewed an infographic intervention (50% increase from baseline knowledge). Mean numeracy score of 44.4% was lower than those in larger German and American populations (68.5% and 64.5%, respectively), but was in an older, less-educated, male population (Galesic et al., 2010). While numeracy skills were evenly distributed among control and intervention groups, separate analysis suggests that scoring well in the numeracy test was the single strongest predictor of being able to correctly interpret assigned information regardless of format. The significantly higher mean numeracy test scores of those who were correct post-intervention illustrate this point. The second strongest predictor was being in possession of at least one educational qualification. In addition, there was a highly significant difference between the mean numeracy scores of those who correctly interpreted assigned information and those who did not. All of this information reinforces the important role of education and numerical literacy in the interpretation of statistical information and the need to further explore means of getting messages to those less well educated where health deficits are greater.

Both infographic interventions improved the probability of assimilating the relationship between cancer risk and old age but this was not statistically significant; likely due to the small numbers in this study. Confidence intervals were too wide to draw any meaningful conclusion about the magnitude of this improvement compared with text control. Interestingly, while participants from both educational groupings had similar knowledge of the association between cancer risk and old age prior to viewing interventions, those with at least one educational qualification seemed to benefit from the information provided (awareness more than tripled post RCT). This contrasts with those with no formal educational qualifications who actually saw a 15% decline in their knowledge. This would seem to indicate that additional information confused or misled the group with no formal educational qualifications. Confusing individuals with visual information is something Arcia et al. (2015) identified as being one of the pitfalls of infographic design—particularly in lower literacy communities.

Qualitative analysis of participants attempts to define the four phrases used in cancer statistics yielded some interesting results. Overall, participants had limited knowledge and understanding of the phrases “cancer incidence” and “cancer prevalence” while understanding of the phrases “cancer risk factor” and “cancer stage” was generally good. Those who were closest to accurately defining the phrases “cancer incidence” and “cancer prevalence” did so using vague numerical terminology. For example, one participant chose to define cancer incidence as “the percentage of population who are diagnosed with cancer” while another wrote that cancer incidence was “the number of people within a certain sample who get cancer.” Multiple participants described cancer prevalence as a “*percentage of population affected by cancer*.” This implies that while exact knowledge of these terms is lacking, there is an understanding that they are statistical terms. The use of named examples (i.e., smoking, alcohol, UV exposure, etc.) while defining “cancer risk factor” shows that there is awareness among the study population of exposures that have a multiplicative effect on cancer risk. One participant in particular gave an extremely cohesive and complete explanation for what a cancer risk factor was—“lifestyle habits such as smoking or drinking, or certain jobs where there are pollutants such as smoke or smoke producing fumes all contribute to the risk of cancer.” Another participant chose to outline less commonly known risk factors for cancer—“exposure to sun without sf cream, unhealthy eating, and lack of exercise.” No participants chose to mention familial risk factors such as genetic mutations. This is likely a consequence of lower educational attainment of participants. Finally, in defining the phrase “cancer stage,” participants used a variety of words and phrases that tell us that there is an appreciation that cancer stage is related to progress and development of cancer. A sizeable minority were also able to further develop this concept to create a semantic link to diagnosis and treatment. For example, one participant wrote that “the early stages where it is localized and operable or later when it has metastasized.” In our opinion, this implies good understanding of what the phrase “cancer stage” means.

### 4.2. Strengths and limitations

The strengths of this study include its novelty, use of an RCT design, and inclusion of participants who are more socially isolated and traditionally more difficult to recruit. The mixed-methods design of this study allows for integration of quantitative and qualitative findings, thus augmenting our understanding of both intervention effects and trial methodology. To our knowledge, this is one of the first trials involving public health infographics and will therefore be of interest to public health decision makers, bioinformatics researchers, and healthcare professionals. Hill et al. (2016) and Zikmund-Fisher et al. (2008) have focused on the benefits of individually tailored visualizations. We hope to add to this body of work by developing evidence-based public health infographics that are capable of explaining statistical information. We know that users of Men Sheds are usually older and sometimes socially isolated. This is a group of people of interest to public health organizations on both sides of the Atlantic (Independent Age, 2017; Steptoe, Shankar, Demakakos, & Wardle, 2013).

Limitations of this study include small sample size and limited generalizability of results. It was our original intention to recruit as many participants as possible to this study but it proved difficult to recruit to this non-incentivized RCT. Recruitment to research studies where there is no financial, material, or therapeutic incentive for participants is known to be challenging (Adams, Caffrey, & McKevitt, 2015). Indeed, a cohort study of 114 large clinical RCT’s found that less than one third (31%) of trials met their initial recruitment target (McDonald et al., 2006). While we are interested in knowing how effective infographics are as a communication medium in older males, results cannot be generalized to other groups such as women. There is evidence to suggest that both genders access healthcare information differently and, in turn, display divergent health seeking behaviors (Bidmon et al., 2015; Stefan, 2015; Thompson et al., 2016). Therefore, it would be beneficial to include women in the next trial phase to determine if any differences exist between genders with regards to numeracy, knowledge of cancer risk in old age, and ability to extract meaning from infographics.

### 4.3. Implications for research and practice

Cancer survival is lower in the UK compared to other socioeconomically similar countries (Coleman et al., 2011). While the reasons for this discrepancy are complex and likely multifactorial, it has been theorized that low awareness of cancer risk and lack of knowledge surrounding cancer may contribute to reduced survival (Forbes et al., 2013). With this in mind, and to the best of our knowledge, this is the first study to collect information about public understanding of some of the words and phrases used in cancer statistics reporting. This should be of interest to public health organizations and cancer registries. While we acknowledge that terms such as “cancer prevalence” and “cancer incidence” are specific and unlikely to be routinely encountered by the general public, we have found low awareness of their definitions among participants who are at a higher risk of cancer. These phrases are mentioned and reported on all of the aforementioned cancer registry websites and because websites such as these are a potential source of knowledge about cancer for the public, they may benefit from being updated accordingly. This includes simplification of how information is presented, removal of terms such as “incidence” or “prevalence,” and provision of visually attractive infographics like the ones used in this study or an interactive digital visualization (Rieger et al., 2018).

## 5. Conclusion

**Figure 3.**
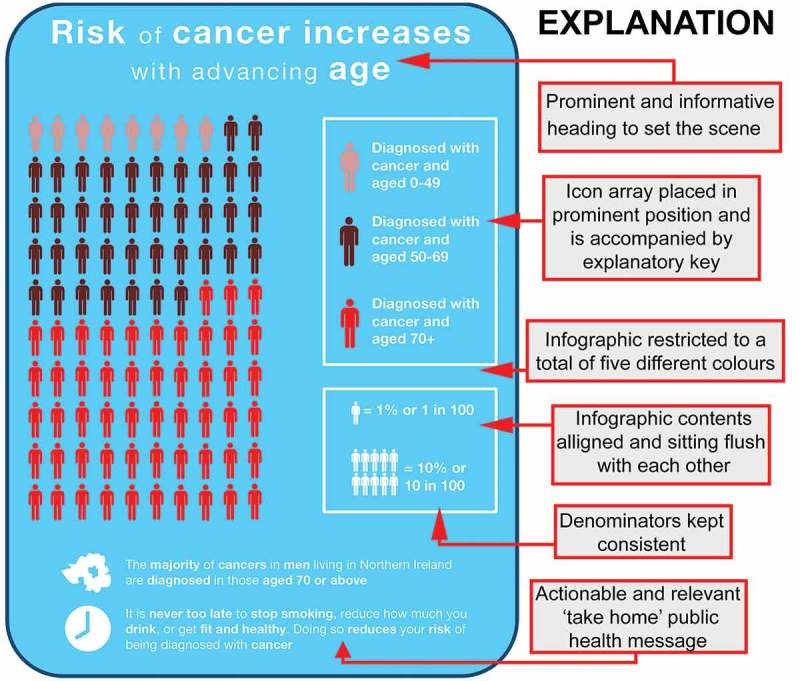
New intervention infographic with icon array, actionable health message, and attractive colors produced according to best practice guidelines.

Infographics can potentially make statistical information more accessible to the general public. We have identified a knowledge gap that will likely benefit from infographics and have piloted a methodology to test this theory. Based on our experience with this initial trial phase, some changes to methodology and recruitment will be beneficial for a future larger scale trial. Potential changes include recruitment of males and females, simplifying to a two-armed RCT design, and creation of a new more colorful icon array intervention with actionable public health message based on Public Health England guidelines (Public Health England, 2017) (see Figure 3). Based on preliminary findings from this study, we have included the word “risk” but omitted any mention of “incidence” or “prevalence” from the new infographic.
