# Supplementary material for: Trial of infographics in Northern Ireland (TINI): Preliminary evaluation and results of a randomized controlled trial comparing infographics with text
Source: Cogent Med. 2018 Jun 11;5(1):1–12. doi: 10.1080/2331205X.2018.1483591 (PMC6002148; doi:10.1080/2331205X.2018.1483591)
Supplement: Supplemental Material [file OAMD_A_1483591_SM0056.zip › Questionnaire.docx]

**Supplementary appendix part I – questionnaire administered prior to RCT**

**QUESTIONNAIRE ID: _______________**

You have fifteen minutes to complete this questionnaire. There are eighteen questions in total.

Q1: **Over the next year, which of these groups of people, if any, do you think is most likely to be diagnosed with cancer?**

☐ 30 year olds

☐ 50 year olds

☐ 70 year olds

☐ People of any age are equally likely to be diagnosed with cancer

Q2: **What do you think the phrase ‘cancer incidence’ means? Please write down your answer in the space provided below. If you don’t know, write down your best guess**

___________________________________________________________________

___________________________________________________________________

Q3: **What do you think the phrase ‘cancer prevalence’ means? Please write down your answer in the space provided below. If you don’t know, write down your best guess**

___________________________________________________________________

___________________________________________________________________

Q4: **What do you think the phrase ‘cancer risk factor’ means? Please write down your answer in the space provided below. If you don’t know, write down your best guess.**

___________________________________________________________________

___________________________________________________________________

Q5: **What do you think the phrase ‘cancer stage’ means? Please write down your answer in the space provided below. If you don’t know, write down your best guess.**

___________________________________________________________________

___________________________________________________________________

Q6: **Imagine that we flip a fair coin 1000 times. What is your best guess about how many times the coin will come up heads in 1000 flips?**

________________ times out of 1000

Q7: **In the bingo lottery, the chance of winning a £10 prize is 1%. What is your best guess about how many people will win a £10 prize if 1000 people each buy a single ticket for the bingo lottery?**

________________ person(s) out of 1000

Q8: **In the Daily Times sweepstakes, the chance of winning a car is 1 in 1000. What percentage of tickets for the Daily Times sweepstakes win a car?**

________________ % of tickets

Q9: **Imagine that we roll a fair 6-sided dice 1000 times. Of 1000 rolls, how many times do you think the dice will come up even (2, 4, or 6)?**

________________ times out of 1000

Q10: **Which of the following numbers represents the biggest risk of getting a disease? 1 in 100, 1 in 1000, or 1 in 10?**

1 in _______

Q11: **Which of the following represents the biggest risk of getting a disease? 1%, 10%, or 5%?**

_______ %

Q12: **If the chance of a person getting a disease is 10%, how many people would be expected to get the disease out of 1000?**

________________ person(s) out of 1000

Q13: **If the chance of getting a disease is 20 out of 100, this would be the same as having what % chance of getting the disease?**

_______ %

Q14: **If person A’s chance of getting a disease is 1 in 100 in 10 years and person B’s risk is double that of A, what is B’s risk?**

_______ in _______

Finally, we would like to find out a little about you. We would like to reassure you that none of the information you provide will reveal your personal identity.

Q15: **What is your age?**

☐ Between 50 and 64

☐ 65 and above

Q16: **What is the highest level of education you have achieved?**

☐ Finished school at or before the age of 15

☐ Completed CSE’s, O-levels, or equivalent

☐ Completed A-levels or equivalent

☐ Completed further education but not a degree

☐ Completed a bachelors degree, masters degree, or doctorate degree

☐ Prefer not to say

☐ Other (please specify)

Q17: **Please indicate which of these best describes your current marital status?**

☐ Married or in a civil partnership

☐ Living with my partner

☐ Single, that is never married and not living with a partner

☐ Divorced or separated and not living with another partner

☐ Widowed and not living with another partner

Q18: **Have you, or any friends or family members that are close to you, ever been diagnosed with cancer?**

☐ Yes, myself

☐ Yes, someone close

☐ Yes, both myself and someone close

☐ Yes, but would prefer not to say who

☐ No

**Supplementary appendix part II – questionnaire administered post RCT**

**QUESTIONNAIRE ID: _______________**

Now that you have viewed the piece of information about cancer, we would like you to answer one final question for us

Q: **Over the next year, which of these groups of people, if any, do you think is most likely to be diagnosed with cancer?**

☐ 30 year olds

☐ 50 year olds

☐ 70 year olds

☐ People of any age are equally likely to be diagnosed with cancer
